# Dietary Ganglioside Reduces Proinflammatory Signaling in the Intestine

**DOI:** 10.1155/2012/280286

**Published:** 2012-02-12

**Authors:** John Janez Miklavcic, Kareena Leanne Schnabl, Vera Christine Mazurak, Alan Bryan Robert Thomson, Michael Thomas Clandinin

**Affiliations:** ^1^4-002 Li Ka Shing Centre for Health Research Innovation, University of Alberta, Edmonton, AB, Canada T6G 2R1; ^2^200, 10150-102 street, Dyna LIFE Diagnostics, Edmonton, AB, Canada T5J 5E2; ^3^Division of Gastroenterology, University of Western Ontario, London, ON, Canada N6A 5A5

## Abstract

Gangliosides are integral to the structure and function of cell membranes. Ganglioside composition of the intestinal brush border and apical surface of the colon influences numerous cell processes including microbial attachment, cell division, differentiation, and signaling. Accelerated catabolism of ganglioside in intestinal disease results in increased proinflammatory signaling. Restoring proper structure and function to the diseased intestine can resolve inflammation, increase resistance to infection, and improve gut integrity to induce remission of conditions like necrotizing enterocolitis (NEC) and Crohn's disease (CD). Maintaining inactive state of disease may be achieved by reducing the rate that gangliosides are degraded or by increasing intake of dietary ganglioside. Collectively, the studies outlined in this paper indicate that the amount of gangliosides GM3 and GD3 in intestinal mucosa is decreased with inflammation, low level of GM3 is associated with higher production of proinflammatory signals, and ganglioside content of intestinal mucosa can be increased by dietary ganglioside.

## 1. Review

**Figure 1:**
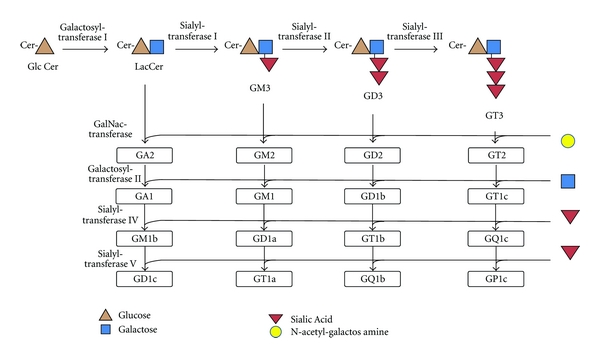
General scheme for ganglioside synthesis. Network of ganglioside synthesis; steps are also reversible. “G” denotes “ganglioside;” “A” denotes “asialo” or lacking sialic acid; “M” denotes “monosicalo,” “D” denotes “disialo;” numbers denote carbohydrate sequence. Adapted from Malisan and Testi [6]. Cer: ceramide; GlcCer: glucosylceramide; LacCer: lactosylceramide; GalNac: N-acetylgalactosamine.

Ganglioside refers to a network of sialylated glycosphingolipids, each with independent biologic properties (Figure 1) [1–3]. Gangliosides are found mainly in the lipid rafts of the intestinal mucosa [4]. Gangliosides consist of a charged, hydrophilic region that protrudes from the membrane surface, and a hydrophobic ceramide anchored in the cell membrane [5].

### 1.1. Ganglioside Synthesis and Degradation

**Figure 2:**
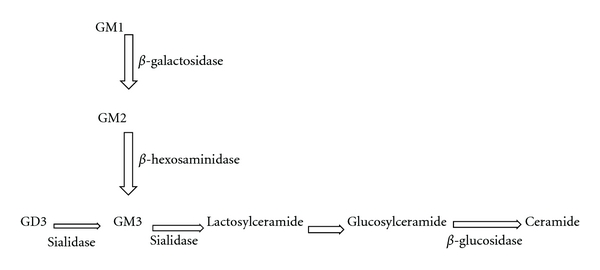
Ganglioside catabolism. Enzyme responsible for catabolic processing step is shown adjacent to arrow. Adapted from Devlin [11].

In mammalian cells, ganglioside synthesis commences with ceramide synthesis in the endoplasmic reticulum [7, 8]. Ceramide is transported to the cytosolic Golgi face for addition of glucose [9]. From this point, sugar moieties and sialic acids are added to form one of several gangliosides. These reactions are accomplished by sialyltransferases, galactosaminyltransferases, and galactosyltransferases on the luminal face of the Golgi complex at controlled rates [10]. Ganglioside catabolism is outlined in Figure 2.

### 1.2. Ganglioside Content and Composition

The amount and content of ganglioside varies among species and in tissues within species [12]. Transcriptional and posttranslational events regulate the amount and content of ganglioside in cells [13]. Ganglioside content is particularly high in the central nervous system, relative to other tissues of the body [14]. The fatty acid tail of ceramide also varies in length within gangliosides [15], as demonstrated in A2780 ovarian carcinoma cells [16]. Variability in sialic acid configuration, oligosaccharide size, and length of ceramide may have consequences that alter ganglioside localization and functionality [17–19]. It is unknown whether many of the health benefits attributed to gangliosides are due to a specific ganglioside species like GM3 or GD3, or whether the fatty acid component of the ceramide tail alters the molecular role of the ganglioside.

## 2. Ganglioside in Diet

In addition to endogenous ganglioside biosynthesis, ganglioside can also be obtained exogenously from diet [20]. The milk fat globule membrane is a biological membrane enriched in ganglioside that protects and stabilizes milk fat in the aqueous phase [21]. Dietary ganglioside intake is very low unless consuming whole-organ foods (i.e., brain), whole milk, buttermilk, or colostrum in high quantities. Several tissues have been shown to incorporate dietary gangliosides. Caco-2 cells incorporate GD3 [22] when provided with ganglioside *in vitro *[23]. Ganglioside uptake also occurs in several tissues *in vivo*. Providing ganglioside in the diet increases ganglioside content in intestinal mucosa [20]. Providing GM3 and GD3 in the diet increased total ganglioside content of epithelial cells within intestine and retina in rats [20, 24]. The estimated average intake of ganglioside in a healthy population is well below levels believed to bear therapeutic benefit [25].

### 2.1. Fates of Dietary Ganglioside

GD3 is specifically localized to the basolateral membrane surface, while GM3 is localized at the brush border membrane of the enterocyte [26]. According to Pagano's vesicle sorting theory [27], absorbed gangliosides have three fates: transport back to the plasma membrane immediately after being endocytosed; endocytosis to the Golgi apparatus for glycosylation to form more complex ganglioside species; transport by the endosome to the lysosome for degradation. Metabolic kinetics of GD3 has been described in depth in Caco-2 cells. GD3 taken up by the brush border membrane is mainly metabolized into new ganglioside species, with smaller portions being retained or transferred, whereas GD3 taken up by the basolateral membrane is not retained or transferred to any significant degree [22]. These observations suggest that each species of ganglioside localizes to particular regions of the enterocyte to carry out specific functions, which depend on site of uptake. There is a gap in understanding of how ganglioside uptake by different cell types and regions of the gut is regulated.

### 2.2. Ganglioside in Intestinal Health

Important observations from animal studies show that inflamed intestinal mucosa has less ganglioside content than healthy intestinal mucosa [28]. Dietary ganglioside is able to replace mucosal gangliosides that are continually degraded in inflammatory states. Moreover, increasing ganglioside content through diet decreases proinflammatory cytokine production in intestinal mucosa [24, 28] and prevents hypoxia-induced bowel necrosis and cell injury in cultured infant bowel [29]. The following section summarizes the different modes of action by which dietary gangliosides promote intestinal health.

## 3. Mechanisms of Action of Ganglioside

### 3.1. Gut Integrity

Previous studies indicate that ganglioside prevents proinflammatory stimuli from disrupting integrity of tight junctions between enterocytes. Feeding ganglioside to rats prevented a lipopolysaccharide- (LPS-) stimulated decrease in cellular tight junction protein occludin [30]. This work indicates that low levels of GM3 in the intestinal mucosa are associated with degradation of tight junction proteins. Improving intestinal integrity is important for management of diarrhea, infection, penetration of allergens, and malnutrition. Guanylate-binding protein-1 (GBP-1) has been recently identified as a marker of intestinal integrity. Downregulation of GBP-1 has been reported to increase permeability and apoptosis of intestinal cells [31]. The effect of ganglioside on GBP-1 stability is currently unknown and is of interest as a potential therapeutic target.

### 3.2. Immune Cell Targeting

Chemokine receptor type 9 (CCR9) enables immune cells to target the gut [32]. While CCR9-positive immune cells are found mainly in small intestine, integrin *α*_4_ *β*_7_-positive cells tend to home to both small intestine and colon [33]. Integrin-mediated binding may be indirectly influenced by ganglioside. In the plasma membrane, gangliosides are known to localize with proteins which bear specific amino acid sequences [34]. GD3 has been shown to cluster with *β*_1_ integrin and affect properties controlled by integrin-mediated signalling [35]. The interactions between gangliosides and integrins have not received much attention, but may provide important insights into homing of immune cells to gut in conditions like inflammatory bowel disease (IBD).

### 3.3. Immune Cell Signaling

Gangliosides are organized into microdomains termed lipid rafts that float freely in the lipid bilayer [36] and serve as organizing centers for assembly of signaling molecules and receptor trafficking [37, 38]. Organization of signaling molecules into lipid rafts is vital for regulation of T-lymphocyte activation pathways that play a major role in pathology of IBD [39, 40]. Disruption of lipid rafts displaces cellular signaling molecules and alters immunoreceptor signal transduction [41–43]. Specifically, sphingolipid depletion inhibits glycophosphatidylinositol-anchored protein trafficking in microdomains [44]. Absence or increased catabolism of ganglioside adversely affects lipid raft trafficking and signaling functions and promotes an inflammatory environment. Gangliosides are imperative for proper structure and function of lipid rafts and dietary ganglioside may disrupt constitutive activation of inflammatory pathways that are hallmark of intestinal disease. 

### 3.4. Proinflammatory Mediators

Inflammation characterizes several chronic diseases including cardiovascular disease, cancer, NEC, and IBD. In culture, inflamed intestinal mucosa has significantly decreased ganglioside content [28]. Changes in ganglioside content and composition also occur in the oncogenic transformation of tissue. Undifferentiated Caco-2 cells have lower total GD3 and polar gangliosides than differentiated Caco-2 model intestinal epithelial cells [45]. It is unknown whether ganglioside catabolism precedes the proinflammatory signals and subsequent inflamed state, or whether inflammation induces ganglioside catabolism. Enrichment of intestinal mucosa with ganglioside causes a reduction in cholesterol content [28]. Cholesterol depletion disrupts membrane microdomain structure and inhibits generation of proinflammatory mediators [46, 47]. In preclinical studies, ganglioside treatment increases ganglioside content and inhibits signals caused by proinflammatory stimuli tumour necrosis factor-*α* and interleukin- (IL-) 1*β* in rats [28]. Similarly, ganglioside reduces IL-6 and IL-8 production in cultured infant bowel when exposed to LPS under hypoxic conditions [29]. Replacing ganglioside that is degraded protects the gut by attenuating proinflammatory signals.

### 3.5. Anti-Inflammatory Mediators

Previous studies have shown enhanced production of IL-10 with dietary ganglioside treatment [30]. IL-10 is an anti-inflammatory cytokine and may be involved in resolution of inflammation. Polyunsaturated docosahexaenoic-acid-derived resolvins and protectins have recently been discovered as having anti-inflammatory properties [48]. Production of resolvin D3 and protectin 1/D1 may be responsible for blocking dextran sodium sulfate-induced colitis in mice [49]. Therefore, resolvins and protectins have been suggested as novel candidates for IBD therapy [50]. Ganglioside in the diet increases the amount of polyunsaturated fat relative to saturated fat in weanling rat intestine [51] and, thus, may enable enhanced production of resolvins and protectins.

### 3.6. Prevention of Infection

**Figure 3:**
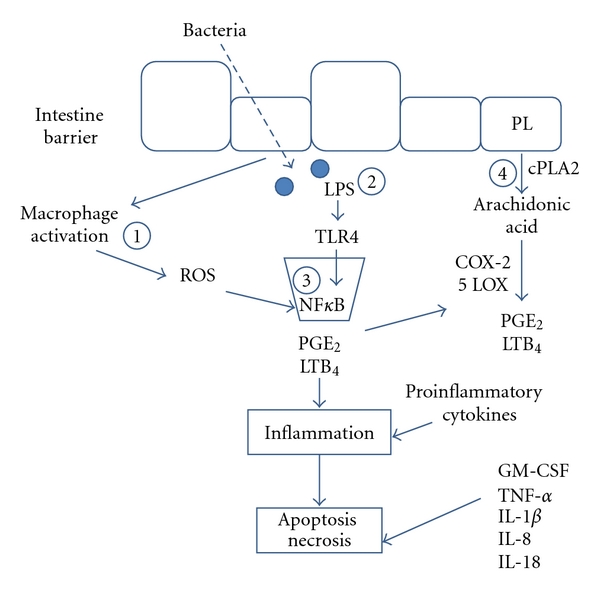
Inflammatory signaling cascade and mechanisms by which ganglioside protects the intestine from inflammation and injury. There are at least four possible mechanisms by which ganglioside protects intestine from injury: (1) gangliosides prevent proliferation, maturation and targeting of immune cells; (2) gangliosides bind enterotoxic LPS and prevent interaction with TLR4; (3) gangliosides inhibit NF*κ*B activation; and (4) gangliosides prevent production of LTB_4_ and PGE_2_. Adapted from Schnabl et al. [29]. COX-2 = cyclooxygenase-2; cPLA2 = cytosolic phospholipase A2; LTB_4_ = leukotriene B_4_; LPS = lipopolysaccharide; 5 LOX = 5 lipoxygenase; PGE_2_ = prostaglandin E_2_; PL = phospholipid; ROS = reactive oxygen species; 1, 2, 3, 4 = steps inhibited by ganglioside; TLR4 = toll-like receptor-4.

Provision of dietary ganglioside known to have antibacterial properties increases the resistance of an individual to negative effects of microbial pathogens. Evidence suggests that patients with IBD may be more prone to infection than healthy individuals [52]. In a Spanish population, mutation in authophagy related 16-like 1 (ATG16L1) is associated with prevalence of CD [53]. ATG16L1 is part of a group of proteins involved in autophagy [54]. Defects in ATG16L1 may allow for infectious organisms to persist, triggering an exacerbated immune response in the gut. Toll-like receptor-4 (TLR4) was found to be higher in intestinal mucosa of children with IBD than healthy controls [55]. Upon stimulation of TLR4 by pathogens or enterotoxins (Figure 3), immune cells produce reactive oxygen species that lead to activation of nuclear transcription factor-kappaB (NF*κ*B) pathway and production of inflammatory mediators. Ganglioside inhibits binding, toxin production, and infectivity of several intestinal pathogens [56, 57], thereby attenuating NF*κ*B inflammatory signaling pathways. Ganglioside may play a critical role in supporting gut health by preventing secondary infection and the associated inflammatory signaling cascade.

### 3.7. NF*κ*B Pathway

CARD15 (nucleotide-oligomerization domain-containing protein 2 (NOD2)) polymorphism has most consistently arisen as a genetic risk factor for CD [58, 59]. The normal function of CARD15 is to suppress NF*κ*B stimulation [60]. Defects in CARD15 allow constitutive activation of NF*κ*B, resulting in chronic inflammation and injury to intestinal mucosa. Nucleotide-oligomerization domain-containing protein 1 (NOD1) is an activator of NF*κ*B and wild-type NOD1 is associated with increased risk of CD [61]. Ganglioside may attenuate NF*κ*B signaling as a previous study showed that GD3 prevented activation of NF*κ*B in mitogen-stimulated T cells [62]. This is particularly important since cyclooxygenase (COX) and lipoxygenase (LOX) enzyme production is increased by stimulation of NF*κ*B pathway [63]. COX and LOX metabolize arachidonic acid (AA) into proinflammatory mediators leukotriene B_4_ (LTB_4_) and prostaglandin E_2_ (PGE_2_). It has been shown that ganglioside prevents production of LTB_4_ and PGE_2_ in infant bowel when cultured with LPS [29]. Ganglioside appears to inhibit production of LTB_4_ and PGE_2_ in intestine by blocking nuclear translocation of NF*κ*B [64].

## 4. IBD Background

Ganglioside has shown therapeutic benefit in models of proinflammatory diseases that have common features with IBD. Collectively known as IBD, CD and ulcerative colitis (UC) severely impede quality of life in afflicted individuals. IBD presents with abdominal pain, gastrointestinal bleeding, diarrhea, weight loss, and malnutrition; all of which negatively impact social and emotional welfare. IBD can be associated with development of joint, liver, and kidney diseases, and an elevated risk of lymphoma and colorectal cancer. Disease management is difficult and may consist of costly drug treatment including steroids, immunosuppressants [65], or antibiotics [66]. Some individuals with IBD do not respond to standard drug treatment, while others experience negative or toxic adverse effects [67]. Administration of prednisolone has been shown as a risk factor for development osteoporosis in older patients with IBD [68]. Severe cases require surgery to remove the affected bowel, and psychological factors including stress may trigger disease flares [69]. The etiologies of CD and UC are poorly understood and there is no cure for IBD. 

### 4.1. IBD Epidemiology

At a rate of 0.60% of the population [70], prevalence of IBD is particularly high in Canada [71] compared to the rest of the world [72–74]. Prevalence of IBD is also high in the United States, where reported incidence is approximately 1.1 million people per year [72]. IBD is a considerable economic burden. In 2008, economic cost per patient with IBD was estimated above $9,000/year in Canada [70]. Another study reported direct healthcare costs greater than $18,000/patient-year in the United States [75]. There is a clear need for knowledge of disease mechanisms to develop novel cost-effective treatment strategies for sustained remission of disease.

### 4.2. IBD Pathology

CD is chronic enteritis that can occur at any site along the gastrointestinal tract. Initial lesions are characterized by tiny mucosal defects termed aphthous ulcers [76]. There is an infiltration of macrophages that release proinflammatory mediators and perpetuate the inflammatory process. This process contributes to development of fibrotic bands and granulomas. Ulcers grow in size and, as submusoca thickens, fistulae may develop. While inflammation associated with CD occurs in a transmural fashion in the colonic wall, UC-associated inflammation is present superficially at the level of mucosa [77]. With respect to immune system involvement, UC is characterized by T_h_1 cells and CD by T_h_2, T_h_17, and cells involved in innate immunity. There is a strong genetic component that contributes to IBD risk, particularly CD [78]. While a number of genes have been linked to aspects of IBD, environment also plays a large role in active disease. IBD rates are very high in industrialized countries like Canada and the USA. Studies have linked urban environment, smoking, diet high in sugar or total fat, antibiotic use in childhood, nonsteroidal anti-inflammatory use, and many other factors to IBD risk [79]. IBD is a multifactorial disorder of complex origin that appears to stem from changes initially occurring at the cell membrane.

### 4.3. Ganglioside in IBD Pathology

Ganglioside species compositions differ among several disease states. For example, Sandhoff's, Gaucher, and Tay Sach's diseases are characterized by abnormal sphingolipid metabolism due to gene deficiencies for catabolic enzymes and accumulation of gangliosides [80]. A few studies have delved into the relationship between genes that regulate ganglioside metabolism and IBD. A genetic variant of lysosomal sialidase is associated with CD [81], but this study did not assess whether ganglioside content correlates with sialidase genotype. Another study showed that there was no difference in *β*-galactosidase enzyme activity between LPS-stimulated mononuclear cells from IBD patients and healthy controls [82]. In the same study, *β*-hexosaminidase enzyme activity was higher in peripheral blood monocytes of patients with IBD than in healthy control subjects when incubated with LPS. Since *β*-hexosaminidase generates GM3 from GM2, accelerated ganglioside catabolism contributes to pathogenesis of IBD.

## 5. Conclusions

IBD is a disorder influenced by many environmental and genetic factors. Signs regularly present in individuals with IBD include chronic inflammation, overactive immune response, and impaired integrity and permeability of gut. While signs and symptoms may subside for short periods of time, recurrence of IBD-related episodes is regular. There is appreciable cost associated with treating IBD. As surgical intervention or drug administration does not result in a cure, there is demand for new treatment initiatives. Emerging evidence shows the critical role of ganglioside in supporting intestinal health. Ganglioside metabolism in the intestinal mucosa is fundamental to the etiology of IBD. Studies show that low levels of ganglioside in the intestinal mucosa are associated with increased levels of inflammatory markers, susceptibility to pathogens, and poor gut integrity. Dietary ganglioside constitutes an exciting new therapeutic agent which targets intestinal cells and associated immune surveillance by interrupting the inflammatory cascade and subsequently alleviating signs and symptoms of inflammatory intestinal diseases. Dietary ganglioside consumption alleviates many of the burdensome processes in models of intestinal disease that are also characteristic of IBD and, thus, may provide benefit to afflicted individuals.

## Abbreviations

AA: Arachidonic acid
ATG16L1: Autophagy-related 16-like 1
CCR9: Chemokine receptor type 9
CD: Crohn's disease
COX: Cyclooxygenase
GBP-1: Guanylate-binding protein-1
IBD: Inflammatory bowel disease
IL: Interleukin
LTB_4_: Leukotriene B_4_
LOX: Lipoxygenase
NEC: Necrotizing enterocolitis
NF*κ*B: Nuclear transcription factor kappaB
NOD1: Nucleotide-binding oligomerization domain-containing protein 1
NOD2: Nucleotide-binding oligomerization domain-containing protein 2
PGE_2_: Prostaglandin E_2_
TLR4: Toll-like receptor-4
UC: Ulcerative colitis.
